# Drug Administration Errors in Hospital Inpatients: A Systematic Review

**DOI:** 10.1371/journal.pone.0068856

**Published:** 2013-06-20

**Authors:** Sarah Berdot, Florence Gillaizeau, Thibaut Caruba, Patrice Prognon, Pierre Durieux, Brigitte Sabatier

**Affiliations:** 1 Department of Pharmacy, Hôpital Européen Georges Pompidou, Assistance Publique - Hôpitaux de Paris, Paris, France; 2 INSERM, UMR S 872, Equipe 22, Centre de Recherche des Cordeliers, Paris, France; 3 INSERM, Centre d’Investigation Épidémiologique 4, Paris, France; 4 Laboratoire Interdisciplinaire de Recherche en Economie de Santé, EA4410, Université Paris Descartes, Sorbonne Paris Cité, Paris, France; 5 Université Paris-Sud 11, Chatenay-Malabry, France; 6 Université Paris Descartes, Sorbonne Paris Cité, Faculté de Médecine, Paris, France; 7 Department of Medical Informatics, Hôpital Européen Georges Pompidou, Assistance Publique - Hôpitaux de Paris, Paris, France; Groningen Research Institute of Pharmacy, United States of America

## Abstract

**Context:**

Drug administration in the hospital setting is the last barrier before a possible error reaches the patient.

**Objectives:**

We aimed to analyze the prevalence and nature of administration error rate detected by the observation method.

**Data Sources:**

Embase, MEDLINE, Cochrane Library from 1966 to December 2011 and reference lists of included studies.

**Study Selection:**

Observational studies, cross-sectional studies, before-and-after studies, and randomized controlled trials that measured the rate of administration errors in inpatients were included.

**Data Extraction:**

Two reviewers (senior pharmacists) independently identified studies for inclusion. One reviewer extracted the data; the second reviewer checked the data. The main outcome was the error rate calculated as being the number of errors without wrong time errors divided by the Total Opportunity for Errors (TOE, sum of the total number of doses ordered plus the unordered doses given), and multiplied by 100. For studies that reported it, clinical impact was reclassified into four categories from fatal to minor or no impact. Due to a large heterogeneity, results were expressed as median values (interquartile range, IQR), according to their study design.

**Results:**

Among 2088 studies, a total of 52 reported TOE. Most of the studies were cross-sectional studies (N=46). The median error rate without wrong time errors for the cross-sectional studies using TOE was 10.5% [IQR: 7.3%-21.7%]. No fatal error was observed and most errors were classified as minor in the 18 studies in which clinical impact was analyzed. We did not find any evidence of publication bias.

**Conclusions:**

Administration errors are frequent among inpatients. The median error rate without wrong time errors for the cross-sectional studies using TOE was about 10%. A standardization of administration error rate using the same denominator (TOE), numerator and types of errors is essential for further publications.

## Introduction

Medication errors are common in the hospital setting and can lead to adverse drug events [1]. In MEDMARX, the errors reported included prescription errors (21%), medication-delivery errors (22%) and administration errors (33%) [2]. Administration, the final step of the medication process, has been less well studied although it directly concerns nurses and patients and is the last barrier before a possible consequence for the patient.

Administration error is defined as a deviation from the physician’s medication order as written on the patient’s chart [3]. The denominator of error rate is generally the Total Opportunity for Errors (TOE) and could be calculated according to 2 definitions but both definitions produce identical results [4]. First, it is defined as the sum of the total number of doses ordered plus the unordered doses given [5]. According to the second definition, it is the sum of the doses given plus the number of omission errors [6,7]. The clinical impact of administration errors can be evaluated using general classification American Society of Health-system Pharmacists (ASHP) [8] or the National Coordinating Council for Medication Error Reporting and Prevention (NCCMERP) [1]. In order to detect drug administration errors, the observation technique gives more efficient, objective, and reliable results than spontaneous reporting or patient chart reviews [3,9]. However, this technique is very time-consuming and cannot be carried out for very long periods of time. Several studies have evaluated administration errors. No systematic reviews using the observation technique have been published so far [10–16]. The Institute of Medicine reported an extensive review of the literature on medication errors and errors rates [17] but did not focus on the observation technique.

The aim of this present systematic review is to systematically analyse the published evidence concerning the prevalence, nature and severity of administration errors in hospitals detected by the observation technique.

## Methods

### Search strategy

The following electronic databases were used: MEDLINE, EMBASE, and the Cochrane Library. Our search concerned published studies in any language from January 1966 up to December 2011 (Figure S1). The reference lists of all selected studies were collected for additional studies.

### Inclusion and exclusion criteria

Studies that reported the detection of medication errors using the observation method [18], and the rate of administration errors among hospital inpatients were included. Cross-sectional, prospective, and intervention studies that evaluated intervention to reduce errors (before-and-after, randomized controlled) were included. Retrospective studies, abstracts, letters to editors, studies in nursing homes, and studies which evaluated errors for only one drug class were excluded. Administration errors could be detected using spontaneous reporting, review of patient charts or observation. Reporting systems suppose that the person reporting is aware that an error was made. Reviewing patient charts is time consuming [19]. Observation method is considered to be the standard for error detection as it yields more objective and reliable results [3,9,20]. Briefly, an observer follows the nurse giving the medications and notes the administration of each dose. The notes are then compared with the prescription. An error is counted when the nurse does not carry out the order accurately. Results are presented using the TOE or the doses observed as the denominator for error rate.

### Study selection and data abstraction

Two reviewers (senior pharmacists) screened the title and abstract of each publication to independently determine eligibility. They screened the full text of the articles and extracted the main outcome independently. One reviewer extracted the data; the second reviewer checked the data. Discrepancies were resolved by consensus between the two reviewers after discussion. If no consensus was achieved, a third researcher arbitrated. Standardised data-extraction forms were used to extract relevant data on inclusion criteria (study design, year and country, study period, hospital setting), methods (sampling, profession of observers, number of observers, definitions used), and the rate, type, and severity of administration errors. Error rates were extracted in the control period for crossover-studies, before intervention for before-and-after studies, and in all groups in the control period in randomized controlled before-after studies (we estimated that during each study, practices may change even in the control groups after intervention). For studies that presented results as the doses observed, authors were contacted for further information so that their results could be expressed as TOE. If the authors had clarified the denominator used, we re-evaluated data with TOE. Types of errors were reclassified according to the ASHP classification [8]: omission error (the failure to administer an ordered dose to a patient before the next scheduled dose, if any), wrong time error (administration of medication outside a predefined time interval from its scheduled administration time (this interval should be established by each individual health care facility)), unauthorized drug error (administration to the patient of medication not authorized by a legitimate prescriber for the patient), wrong dose error (administration to the patient of a dose that is greater than or less than the amount ordered by the prescriber or administration of duplicate doses to the patient, i.e., one or more dosage units in addition to those that were ordered), wrong dosage-form error (administration to the patient of a drug product in a different dosage form than ordered by the prescriber), wrong drug-preparation error (drug product incorrectly formulated or manipulated before administration), wrong administration technique error (inappropriate procedure or improper technique in the administration of a drug), deteriorated drug error (use of expired drugs or improperly stored drugs) and other medication error (including any drug administration errors not fitting into the predefined categories). The ASHP classification did not specify the time limits used to determine a wrong time error: generally 30 or 60 minutes before or after the scheduled prescription time [21]. Authors [3] recommended that studies on medication errors should report both the errors rate with and without timing error as clinicians often consider wrong time error to be minor. Studies that reported clinical impact were analyzed and impact was reclassified by the two reviewers into 4 main categories (fatal, life-threatening, significant, minor or no impact) when possible. Discrepancies were resolved by consensus between the two reviewers after discussion.

### Risk of bias or quality of reporting

We assessed the quality of reporting of the studies using an evaluation scale adapted from the STROBE Statement checklist [22]. Each item was filled by one reviewer and was validated by the second reviewer.

### Main outcomes

The main outcome was the error rate without wrong time errors and measured at the study level. The secondary outcomes were the error rate including wrong time errors at 60 minutes and clinical impact of errors.

Studies were analyzed separately for administration doses observed and TOE, and according to study designs except for a subgroup exploratory analysis. Egger et al. recommend the results from randomized control trials (RCTs) and non-randomized control trials (non-RCTs) to be stratified to explore potential heterogeneity due to study designs [23].

The global error rate was calculated for each study as the number of errors (or the number of administration with at least one error) divided by the TOE or the doses observed (multiplied by 100). We chose to present the results with the number of errors as numerator, as the studies presenting the number of administration also presented the number of errors. When the number of errors exceed the number of observations (Zribi Triki [24], Gokhman [25]), we modified the numerator to be equal to the denominator. For each study (when available), we also calculated the error rate by type of error using the number of errors of the considered type in the numerator (and the TOE or doses observed in denominator). In a subgroup exploratory analysis, we compared the error rates in studies evaluating injectable drugs only and oral drugs only; and error rates in intensive care units only versus other units.

### Statistical analyses

Given the heterogeneity within the review, we did not undertake a formal meta-analysis. The results were reported using the median error rate across the studies with interquartile range (IQR). The median for the global error rate corresponds to the error rate above which (or equivalently below which) 50% of the studies lie for their observed global error rate. The median for the error rate for a particular type corresponds to the error rate above which (or equivalently below which) 50% of the studies lie for their observed error rate for this particular type (considering only studies reporting this type of error). Heterogeneity was presented using the Cochran Q Chi-squared test and Higgins’ and Thompsons’ I^2^ statistic. A value of 0% indicates no observed heterogeneity, and larger values show increasing heterogeneity. I^2^ values greater than 75% with low P values from the Cochran Q test were considered as indicating substantial heterogeneity. To study the publication bias, we plotted the study precision (within-study standard error) against the log-odds of error rate without wrong time errors (funnel plot). If studies with large proportions of errors were less likely to be published, the funnel plot would appear asymmetric about the vertical. Funnel plot asymmetry was assessed using Egger’s test [26]. Under the null hypothesis of no small study effects, the regression line of the effect against its standard error would be vertical.

The software R was used for forest plots (version 2.12.0); the software Stata was used for funnel plots (StataCorp. 2009. Stata Statistical Software: Release 11. College Station, TX: StataCorp LP; commands “metareg” and “metabias”); and the software SAS was used for descriptive statistics (version 9.2; SAS Institute Inc., Cary, NC, USA). The analysis conformed to the PRISMA checklist (Table S1).

## Results

**Figure 1:**
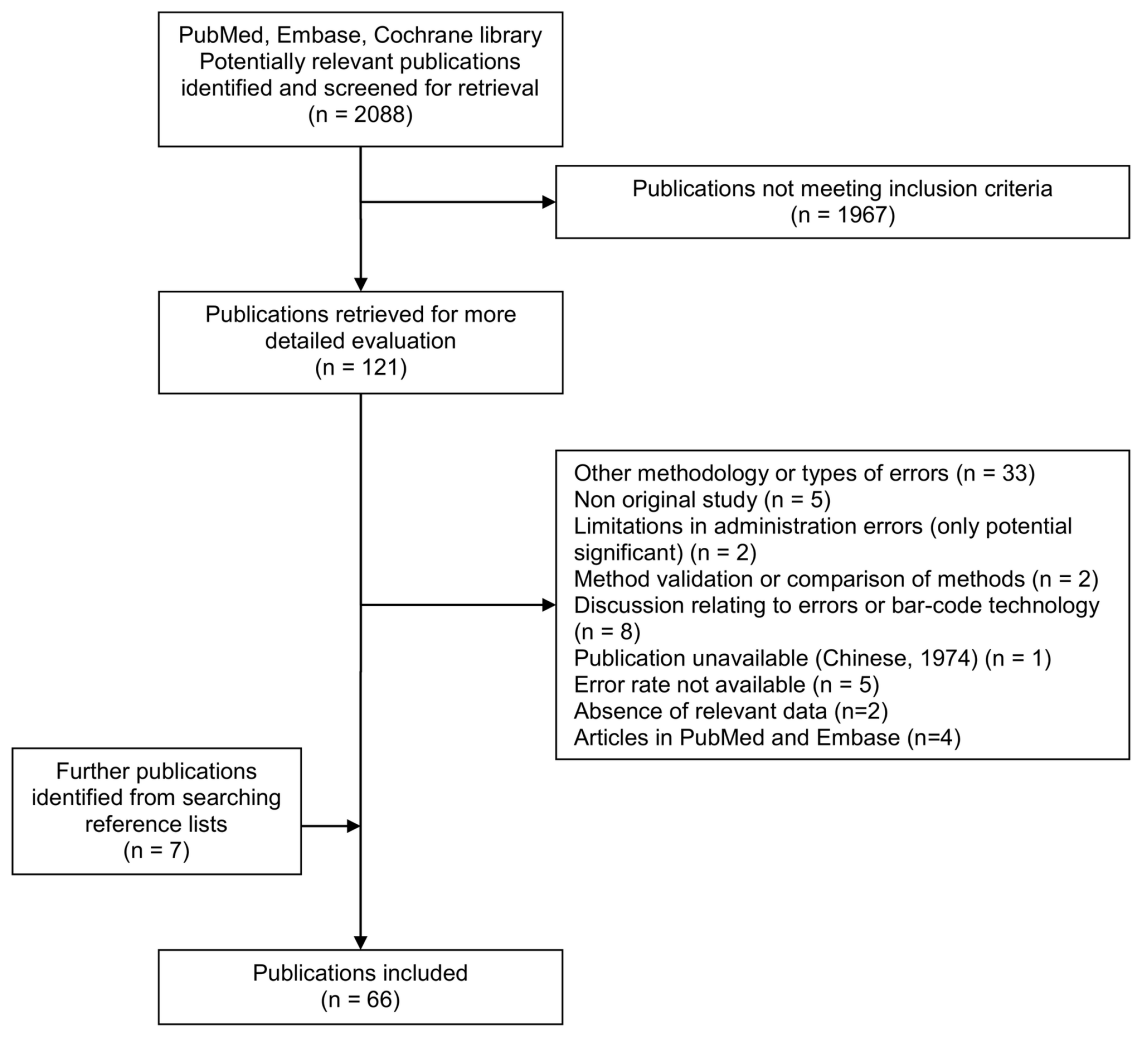
Flow diagram of the screening process.

The electronic search initially identified 2088 publications. After screening titles and abstracts, 1967 publications did not meet the inclusion criteria. The remaining 121 publications were obtained in full text and assessed for suitability. Searching of the reference lists of the included publications identified one other study, published before 1966, that was eligible as well as six other publications. Overall, 66 publications were included [6,7,10–16,18,24,25,27–80] (Figure 1).

### Characteristics of included studies

The details of the 66 studies are presented in Table S2 (studies classified by denominator). They were cross-sectional studies (N=46, 70%), before-and-after studies (N=16, 24%), RCTs (N=2, 3%), controlled before-and-after study (CBAs) (N=1, 2%), and cross-over study (N=1, 2%). None of the studies met all 15 quality of reporting assessment criteria (Table 1).

**Table 1:** Number of studies with information reported in the text.

General items	Items	Number of studies describing the item (%) N=66
Nurse	Selection method of observed nurses	24 (36.4)
	Number of observations per nurse	5 (7.6)
	Selection method of observation period	12 (18.2)
	Number of observed nurses	28 (42.4)
	Total number of nurses in the ward or during the observation period	12 (18.2)
	Nurse practice experience	23 (34.8)
	Nurse aware (or not) of the aim of the study	49 (74.2)
Route of drug administration	IV, oral or both	59 (89.4)
Patients	Number of patients	34 (51.5)
Observation method	Error definition	57 (86.4)
	Type and number of observers	62 (93.9)
	Training of observers	40 (60.6)
	Classification of administration errors defined	57 (86.4)
	DO or TOE^a^ identified	66 (100.0)
	Clinical impact evaluated	32 (48.5)

a DO: doses observed, TOE: Total Opportunity for Errors. Error rate was reported to be calculated using doses observed in 23 publications. After contacting the authors, the denominator definition was confirmed for 5 studies, was declared to be TOE for 9 studies, and remained unanswered for 9 studies. Overall, we identified 14 publications using the doses observed to calculate the error rate and 52 using TOE.

### Types of errors

For 23 publications, the doses observed was used as denominator to calculate the error. After contacting the authors, the denominator definition was confirmed for 5 studies, was declared to be TOE for 9 studies, and remained unanswered for 9 studies. Overall, we analyzed 14 publications using the doses observed and 52 using TOE. Types of errors could not be extracted for 4 studies: Greengold [45] (not including wrong time errors), Dean [37] (not including wrong time errors), Hynniman [6] and Schnell [7] for which only the number of wrong time errors could be extracted. Wrong time errors and wrong drug-preparation errors were the most frequent types of errors (Table 2).

**Table 2:** Types and rates of administration errors.

Types of errors (ASHP)^a^	Number of studies^c^ (TOE^b^)	N (TOE)	Median Rate (%) [Q1-Q3]^d^ (TOE)	Number of studies^c^ (DO^b^)	N (DO)	Median Rate (%) [Q1-Q3]^d^ (DO)
omission	42	69623	1.6 [0.8-4.1]	9	4534	1.6 [0.8-2.8]
wrong-time error^e^	41	86525	4.4 [1.3-16.1]	8	9839	7.2 [1.8-12.6]
wrong-time error (30mn)	7	5908	26.9 [9.2-31.4]	1	572	1.57
wrong-time error (60mn)	22	44497	5.4 [1.9-15.0]	4	7412	8.6 [7.2-13.0]
unauthorized drug error	42	72339	0.3 [0.1-0.8]	12	11576	0.7 [0.2-1.6]
wrong dose error	47	78164	1.4 [0.7-3.4]	12	11576	3.2 [2.6-5.2]
wrong dosage-form error	31	54036	0.1 [0.0-0.3]	6	9884	0.5 [0.2-0.8]
wrong drug-preparation error	30	49912	2.1 [0.1-6.2]	8	6893	8.6 [3.3-30.3]
wrong administration technique error	43	74820	1.2 [0.03-3.5]	13	12261	4.1 [1.8-14.6]
deteriorated drug error	19	33161	0.1 [0.0-0.8]	2	317	0.7 [0.0-1.4]
other medication error	24	50402	1.4 [0.4-3.5]	3	1167	0.8 [0.6-3.3]

^a^ Types of errors could not be extracted for 4 studies (Greengold [45], Dean [37] and Hynniman [6], Schnell [7] except for wrong time errors).

b DO: doses observed, TOE: Total Opportunity for Errors.

c Number of studies considering the error type (among the 52 studies with TOE and 14 studies with doses observed).

e Wrong time errors evaluated for 49 studies (41 TOE and 8 studies using doses observed). Among these 49 studies, 15 did not specify the wrong time delay and therefore were not analysed.

d Median rate for the studies considering the error type [First Quartile – Third Quartile]. Unit of analysis for the median was the study that is 50% of the studies had an observed error rate greater (or lower) than the median rate reported.

### Error unit

#### Error rate without wrong time errors

**Figure 2:**
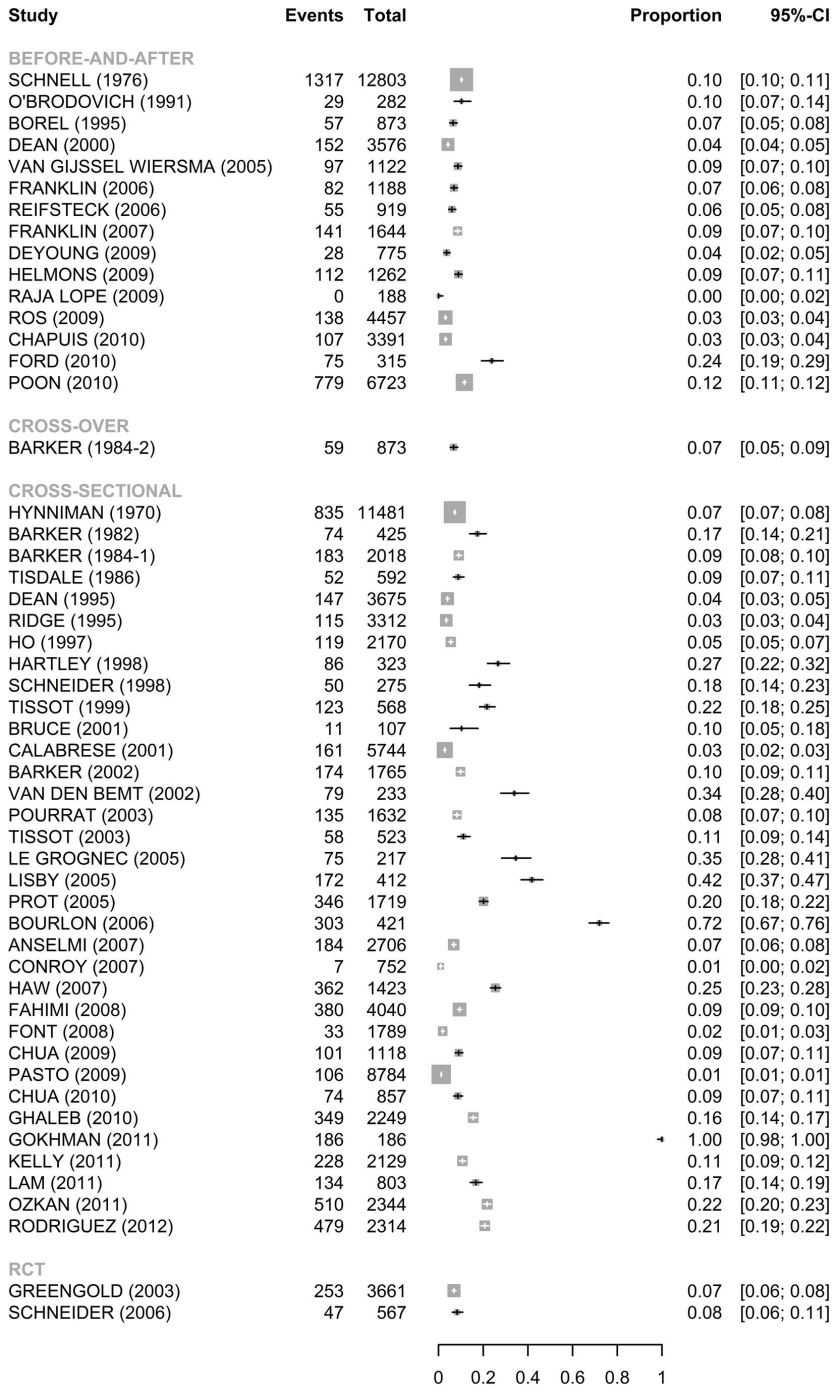
Forest plots for error rate (95%CI) without wrong time errors (TOE).

For the publications using TOE (Figure 2), the median error rates without wrong time errors were 10.5% [IQR: 7.3%-21.7%] for the 34 cross-sectional studies, 6.9% [IQR: 3.6%–10.3%] for the 15 before-and-after studies, 7.5% and 8.3% for the 2 RCT studies, and 6.8% for the cross-over study. There was a considerable heterogeneity among the 34 cross-sectional studies (Q=6205.2, *p*<0.001, I^2^= 99.5%) and the 15 before-and-after studies (Q=785.0, *p*<0.001, I^2^= 98.2%), whereas the heterogeneity was not as apparent among the 2 RCT studies (Q=1.5, *p*=0.23, I^2^= 30.9%). There was no obvious asymmetry in the funnel plot suggesting no evidence of publication bias (Egger’s test: *p*=0.57) (Figure S2/A).

**Figure 3:**
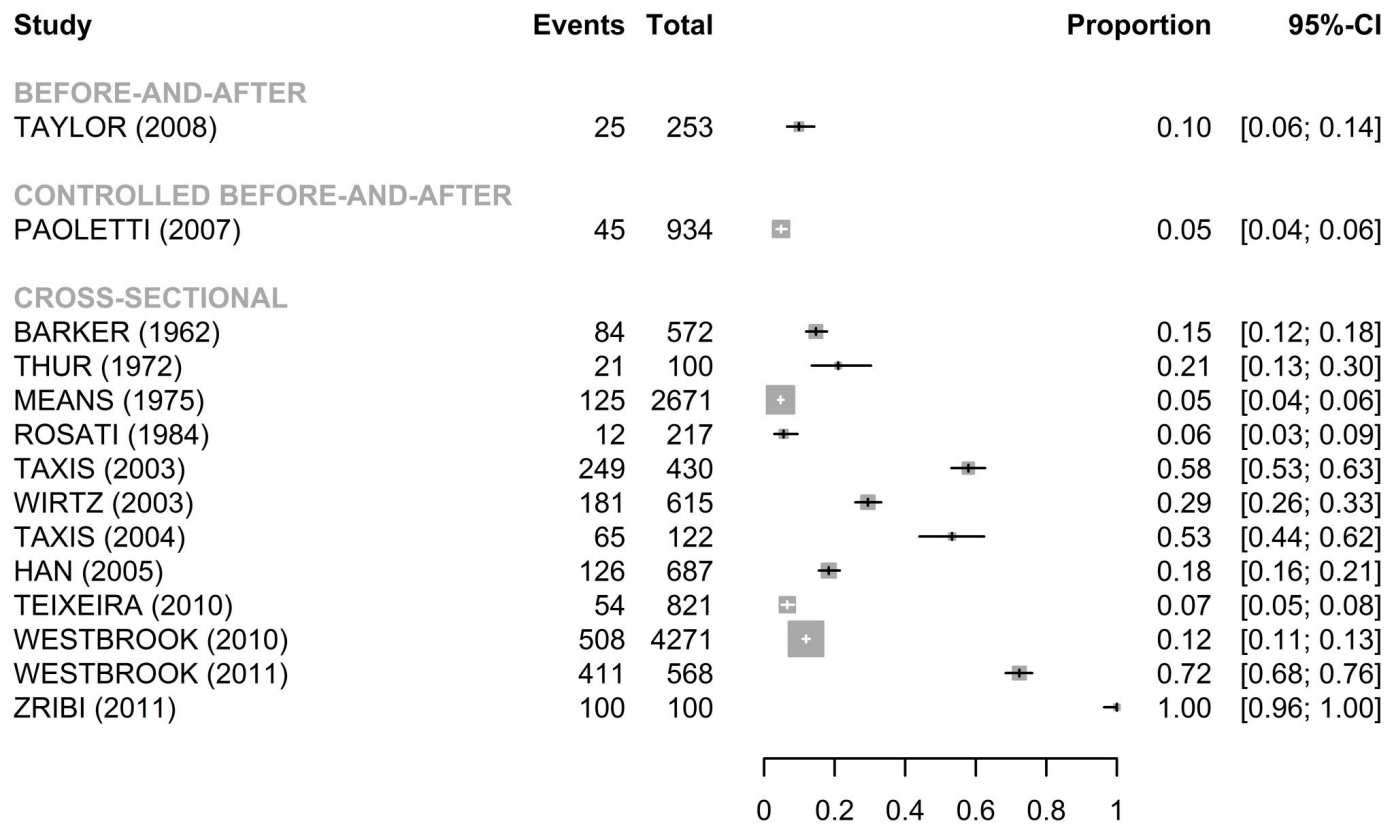
Forest plots for error rate (95%CI) without wrong time errors (doses observed).

For the publications using doses observed (Figure 3), the median error rates without wrong time errors were 19.7% [IQR: 9.2%-55.6%] for the 12 cross-sectional studies, 9.9% for the before-and-after study, and 4.8% for the controlled before-and-after study. There was a considerable heterogeneity among the 12 cross-sectional studies (Q=2386.4, *p*<0.001, I^2^= 99.5%). There was no obvious asymmetry in the funnel plot suggesting no evidence of publication bias (Egger’s test: *p*=0.84) (Figure S2/B).

Wrong time errors were analysed in 49 studies of the 66 studies. Among these studies, 26 studies defined the wrong time delay as 60 minutes, 8 as 30 minutes and the 15 other studies (Schnell [7], Hynniman [6], Borel [31], Bourlon [32], Conroy [36], Haw [48], Font Noguera [40], Ghaleb [44], Gokhman [25], Rodriguez-González [65], Schneider [69], Helmons [49], Paoletti [57], Teixeira [72], Zribi Triki [24]) did not specify the wrong time delay. These 15 studies were not included in the further analyses.

#### Error rate including wrong time errors at 60 minutes

**Figure 4:**
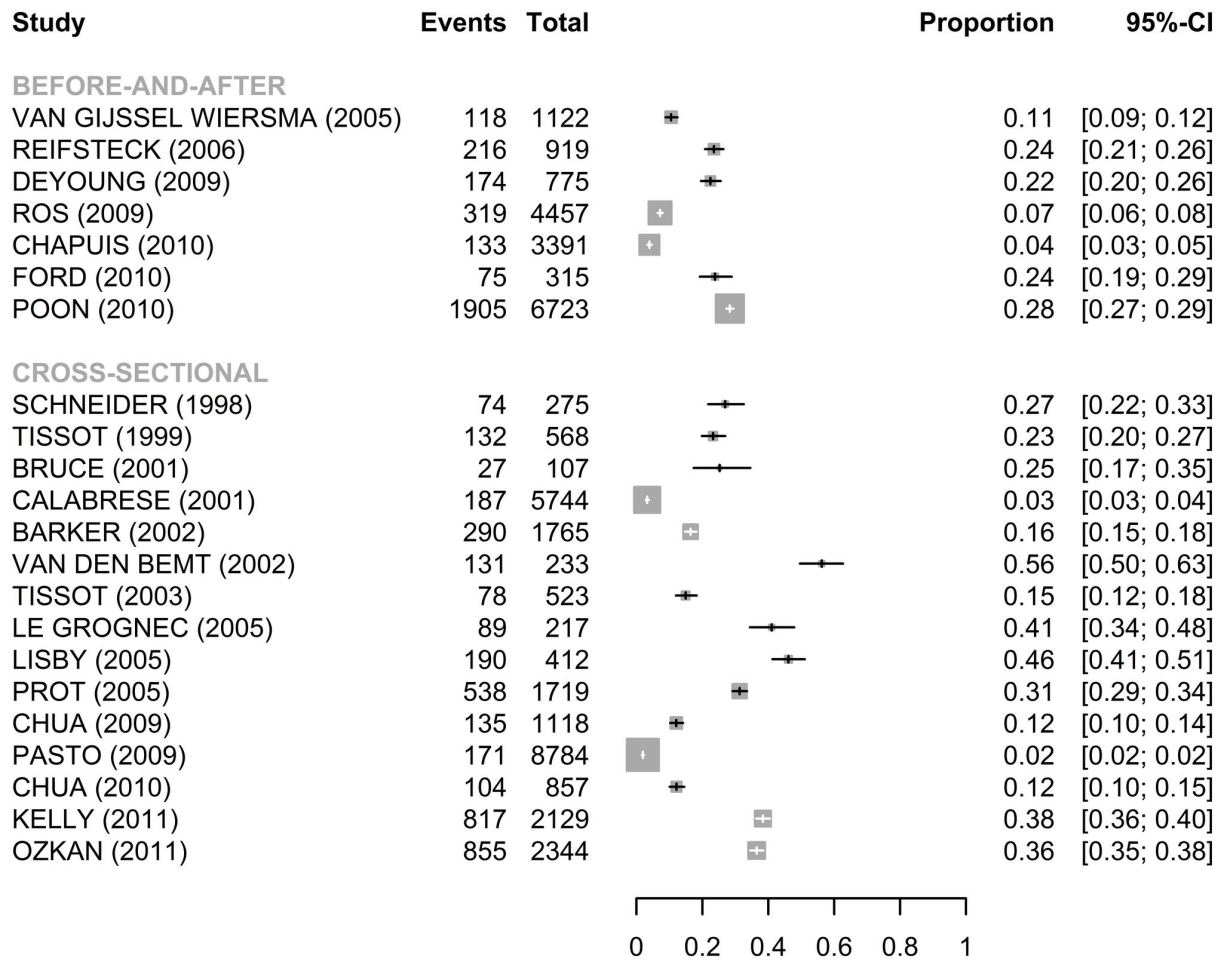
Forest plots for error rate (95%CI) including wrong time errors at 60 minutes (TOE).

**Figure 5:**
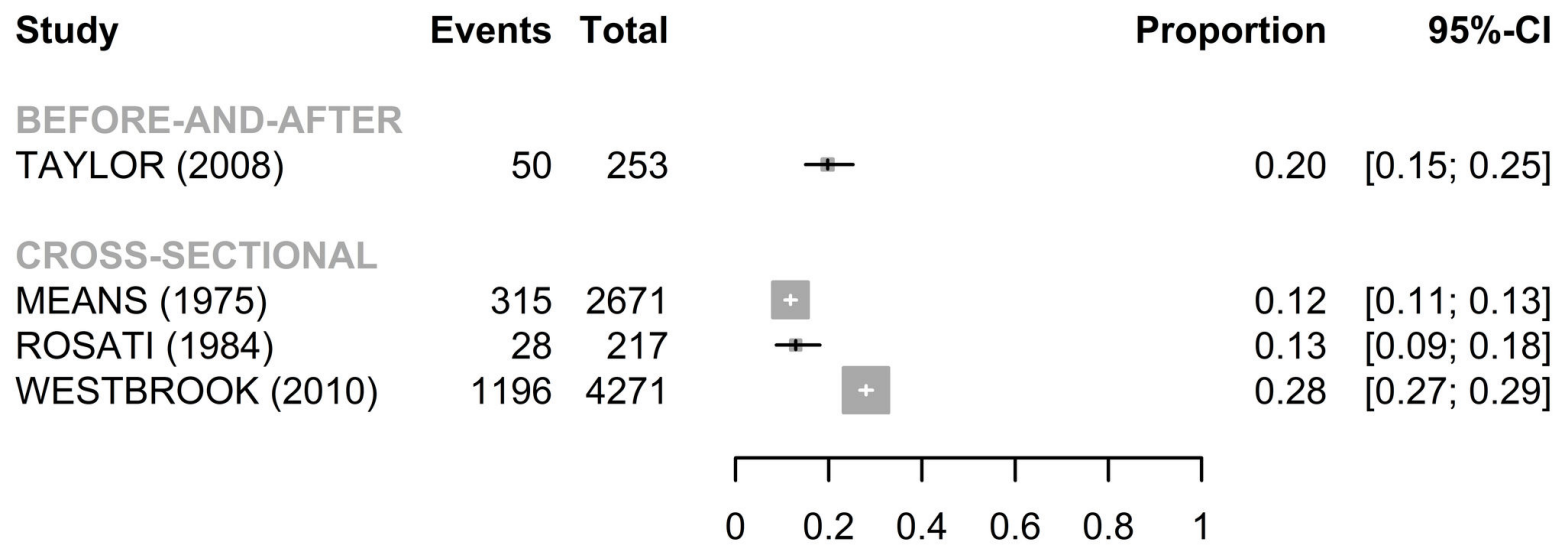
Forest plots for error rate (95%CI) including wrong time errors at 60 minutes (doses observed).

For the publications using TOE (Figure 4), the median error rates including wrong time errors were respectively 25.2% [IQR: 12.1%-38.4%] (N=15) for the cross-sectional studies, and 22.5% [IQR: 7.2%–23.8%] (N=7) for the before-and-after studies. For the publications using doses observed (Figure 5), the median error rates including wrong time errors were respectively 11.8%, 12.9% and 28.0 for the 3 cross-sectional studies, and 19.8% for the before-and-after study. Analyses with wrong time delay at 30 minutes are on demand.

#### Exploratory analysis for studies using TOE

For this exploratory analysis, we excluded studies evaluating both injectable and oral drugs, and studies where the type of unit was unknown or where the units were mixed (medical, surgical and intensive care). Concerning the route of administration, 4 studies evaluated only injectable drugs. The median error rate without wrong time errors was 9.8% [min-max: 6.8%-26.6%]. For the 5 studies evaluating only oral drugs, the median error rate without wrong time errors was 5.5% [min-max: 4.0%-16.7%]. Concerning the characteristics of the unit, 8 studies evaluated error rate in intensive care units. The median error rate without wrong time errors was 9.1% [IQR: 3.4%-19.9%]. For the 33 studies evaluating errors in other units, the median error rate without wrong time errors was 8.6% [IQR: 6.8%-16.7%].

### Clinical impact

Clinical impact of errors was specified in 48% of the studies (32 studies). For 18 studies, we could reclassify the data in 4 main categories (fatal, life-threatening, significant, minor, or no impact) (Table S3) [11,12,14,15,35,48,52,53,58–60,65,70,75–78,80]. No fatal error was observed. In the 14 other studies, most of errors were classified as minor.

## Discussion

We found 66 studies which reported the rate of administration errors detected by the observation method. These studies presented different study designs and did not report the error rate using the same unit for the denominator (TOE or doses observed). In addition these studies did not consider all 9 types of errors from the ASHP, such as wrong time errors. We therefore did not perform a formal meta-analysis. Most of the studies were cross-sectional studies (N=46, 70%). The median error rates without wrong time errors reached 10.5% for the 34 cross-sectional studies using TOE and 19.7% for the 12 cross-sectional studies using doses observed. Median error rates tended to be lower for interventional studies (RCT studies, before-and-after studies, cross-over study).

Clinical impact was evaluated for 32 studies (48%) and classification was heterogeneous. No fatal error was observed and most of the errors observed were minor.

To our knowledge, this is the first systematic review of administration errors detected by the observation method. In a review, Ghaleb [81] identified 32 studies reporting the incidence of medication error, of which 8 concerned drug-administration errors detected by observation. Error rates varied from 0.6% to 27% of administrations but the study selection was not exhaustive. Miller [82] conducted a systematic literature review on medication errors for children between 2000 and 2005; 14 studies evaluated administration errors and none of them used the observation method to determine error. The only study [83] using TOE for error rate showed an administration error rate of 23.5%.

The extreme heterogeneity of studies definitions (definition of error rate with number of errors or number of administrations with at least one error, TOE or doses observed, error classification) is well known [17]. That is why we focused on one error identification methodology (direct observation). But heterogeneity was also found within this methodology. A major source of the high heterogeneity between studies could be the calculation of error rate. The types of errors considered in many studies did not use the ASHP classification and these studies therefore did not consider all possible types of errors. As a consequence, the definition of error rates (numerator and denominator) varied according to the study, in particular the definition of wrong time error. However, the definition of the error rate without wrong time errors remained heterogeneous among the studies because they did not consider all other types of errors. These other types of errors seemed to be less reported because they were less frequent. For example, deteriorated drug errors (use of expired drugs or improperly stored drugs) were reported in only 28% of studies using TOE but the median error rate in these studies was only 0.1%.

Our review has some limitations.

We selected studies in which the observation method, either disguised or undisguised, was used to detect drug errors. The disguised observation may be the gold standard to evaluate error rate. However, the information whether the observation was disguised or not was available in only 49 studies (74% of the studies included). There was no evidence of difference between the two types of observation method.

Important characteristics such as the type of units and the route of administration of drugs (injectable and oral) could only be evaluated in an exploratory analysis as the number of studies was too small. However, there did not seem to have difference in the error rates in different subgroups.

We used an evaluation scale adapted from the STROBE Statement checklist [22] to evaluate whether information was reported. However, no conclusion was made as to whether it was done appropriately in the study. We investigated the presence of publication bias using funnel plots. These analyses indicated there was no evidence of publication bias, despite that publication bias in a review may not always result in an asymmetrical funnel plot [84].

The calculation of error rate was not homogenous: some authors used the number of errors and others used the number of administrations with at least one error. We only presented the results with the first definition (number of errors) as most of the studies presented this type of rate. Authors described the number of errors by type and calculated the error rate without wrong time error by subtracting the number of wrong time errors from the total number of errors. When the number of errors exceeded the number of observations, we evaluated that the error rate reached 100%. The error rate appeared high even when it did not include wrong time errors. Information on how serious these errors were and the rigor of error definition was not available in all of the publications that we selected.

Standardization of administration error rate using the same denominator (TOE) and types of errors remains essential for further publications. To calculate an error rate, we recommend excluding some types of errors, and reporting the number of administrations (TOE) with at least one error and the number of errors and their types according to ASHP classification (9 types). We also advise reporting of the time limits used to determine wrong time errors. If the study implicates an evaluation of medication process, we recommend distinguishing the error rate per process (order, dispensing, and preparation/administration). Finally, the characteristics of hospital need to be presented (country, types of units, delivery system, characteristics of nurses observed).

We did not evaluate interventions to improve administration errors which are important pieces of information for the clinician. The Canadian Agency for Drugs and Technologies in Health [85] and the US Agency for Healthcare Research and Quality [86] recommend the use of technologies to reduce administration errors. This may include improving the dispensing process with an automated bedside dispensing machine or unit-dose dispensing, or improving the administration process by using dedicated medication nurses with specific training, education on drug safety, medication charts, or a bar-code medication administration system. The impact of these technologies needs to be more evaluated.

In summary, administration errors are frequent among inpatients. The median error rate without wrong time errors for the cross-sectional studies using TOE was about 10%. No fatal error was observed and most errors were classified as minor in the 18 studies in which clinical impact was analyzed. We did not find any evidence of publication bias. A standardization of administration error rate using the same denominator (TOE), numerator and types of errors is essential for further publications.

## Supporting Information

Figure S1 Search strategy in Medline (A), Embase (B) and Cochrane library (C). (TIFF)

Figure S2 Funnel plots for log-odds of error rate without wrong time errors for TOE (A) and doses observed (B). Points corresponds to estimates from each studies and dash lines give to the prediction interval of standard errors. The p-value from the Egger’s test indicated that there was no evidence of publication bias (asymmetry).
(TIF)

Table S1 Prisma checklist. (DOC)

Table S2 Characteristics of included studies. (DOC)

Table S3 Clinical impact of administration errors. (DOC)

## References

[1] About (2008) Medication Errors. The National Coordinating Council for Medication Error Reporting and Prevention (NCC MERP). Available: http://www.nccmerp.org/aboutMedErrors.html . Accessed 2009 Sep 12 10.1016/s1553-7250(08)34091-4 19119722

[2] Hicks RW, Cousins DD, Williams RL (2004) Selected medication-error data from USP’s MEDMARX program for 2002. Am J Health Syst Pharm 61: 993-1000. PubMed: 15160775. 15160775 10.1093/ajhp/61.10.993

[3] Allan EL, Barker KN (1990) Fundamentals of medication error research. Am J Hosp Pharm 47: 555-571. PubMed: 2180287. 2180287

[4] Allan BL (1987) Calculating medication error rates. Am J Hosp Pharm 44: 1044, 1046. PubMed: 3605113. 3605113

[5] Barker KN (1969) The effects of an experimental medication system on medication errors and costs. I. Introduction and errors study. Am J Hosp Pharm 26: 324-333. PubMed: 5797287. 5797287

[6] Hynniman CE, Conrad WF, Urch WA, Rudnick BR, Parker PF (1970) A comparison of medication errors under the University of Kentucky unit dose system and traditional drug distribution systems in four hospitals. Am J Hosp Pharm 27: 802-814. PubMed: 5473470. 5473470

[7] Schnell BR (1976) A study of unit-dose drug distribution in four Canadian hospitals. Can J Hosp Pharm 29: 85-90. PubMed: 1024023. 1024023

[8] (1993) ASHP guidelines on preventing medication errors in hospitals. Am J Hosp Pharm 50: 305-314. PubMed: 8480790. 8480790

[9] Flynn EA, Barker KN, Pepper GA, Bates DW, Mikeal RL (2002) Comparison of methods for detecting medication errors in 36 hospitals and skilled-nursing facilities. Am J Health Syst Pharm 59: 436-446. PubMed: 11887410. 11887410 10.1093/ajhp/59.5.436

[10] Anselmi ML, Peduzzi M, Dos Santos CB (2007) Errors in the administration of intravenous medication in Brazilian hospitals. J Clin Nurs 16: 1839-1847. doi:10.1111/j.1365-2702.2007.01834.x. PubMed: 17880472. 17880472 10.1111/j.1365-2702.2007.01834.x

[11] Calabrese AD, Erstad BL, Brandl K, Barletta JF, Kane SL et al. (2001) Medication administration errors in adult patients in the ICU. Intensive Care Med 27: 1592-1598. doi:10.1007/s001340101065. PubMed: 11685299. 11685299 10.1007/s001340101065

[12] Chua SS, Tea MH, Rahman MH (2009) An observational study of drug administration errors in a Malaysian hospital. J Clin Pharm Ther 34: 215-223. doi:10.1111/j.1365-2710.2008.00997.x. PubMed: 19250142. 19250142 10.1111/j.1365-2710.2008.00997.x

[13] Dean BS, Allan EL, Barber ND, Barker KN (1995) Comparison of medication errors in an American and a British hospital. Am J Health Syst Pharm 52: 2543-2549. PubMed: 8590237. 8590237 10.1093/ajhp/52.22.2543

[14] Lisby M, Nielsen LP, Mainz J (2005) Errors in the medication process: frequency, type, and potential clinical consequences. Int J Qual Health Care 17: 15-22. doi:10.1093/intqhc/mzi015. PubMed: 15668306. 15668306 10.1093/intqhc/mzi015

[15] Taxis K, Barber N (2003) Ethnographic study of incidence and severity of intravenous drug errors. BMJ 326: 684. doi:10.1136/bmj.326.7391.684. PubMed: 12663404. 12663404 10.1136/bmj.326.7391.684 PMC152365

[16] Wirtz V, Taxis K, Barber ND (2003) An observational study of intravenous medication errors in the United Kingdom and in Germany. Pharm World Sci 25: 104-111. doi:10.1023/A:1024009000113. PubMed: 12840963. 12840963 10.1023/a:1024009000113

[17] Aspden P, Wolcott JA, Bootman JL, Cronenwett LR (2007) Preventing medication errors: Quality Chasm Series. Available: http://www.nap.edu/catalog/11623.html . Accessed 2012 Feb

[18] Barker KN, McConnell WE (1962) The problems of detecting medication errors in hospitals. Am J Hosp Pharm 19: 361-369.

[19] Montesi G, Lechi A (2009) Prevention of medication errors: detection and audit. Br J Clin Pharmacol 67: 651-655. doi:10.1111/j.1365-2125.2009.03422.x. PubMed: 19594533. 19594533 10.1111/j.1365-2125.2009.03422.x PMC2723204

[20] Meyer-Massetti C, Cheng CM, Schwappach DL, Paulsen L, Ide B et al. (2011) Systematic review of medication safety assessment methods. Am J Health Syst Pharm 68: 227-240. doi:10.2146/ajhp100019. PubMed: 21258028. 21258028 10.2146/ajhp100019

[21] Pepper GA (1995) Errors in drug administration by nurses. Am J Health Syst Pharm 52: 390-395. PubMed: 7757866. 7757866 10.1093/ajhp/52.4.390

[22] University of Bern (2009) STROBE Statement. Available: http://www.strobe-statement.org/. Accessed 2009 December 15.

[23] Egger M, Davey Smith G, Altman DG (2001) Systematic reviews in health care: meta-analysis in context. London: BMJ Publishing House Books.

[24] Zribi Triki E, Belmabrouk R, Keskes H, Sfar S (2011) [Errors in preparation and administration of parenteral drugs in a Tunisian hospital: a prospective study]. Le Pharmacien Hospitalier et Clinicien 46: 226-230. doi:10.1016/j.phclin.2011.05.022.

[25] Gokhman R, Seybert AL, Phrampus P, Darby J, Kane-Gill SL (2012) Medication errors during medical emergencies in a large, tertiary care, academic medical center. Resuscitation 83: 482-487. PubMed: 22001000. 22001000 10.1016/j.resuscitation.2011.10.001

[26] Egger M, Davey Smith G, Schneider M, Minder C (1997) Bias in meta-analysis detected by a simple, graphical test. BMJ 315: 629-634. doi:10.1136/bmj.315.7109.629. PubMed: 9310563. 9310563 10.1136/bmj.315.7109.629 PMC2127453

[27] Barker KN, Flynn EA, Pepper GA, Bates DW, Mikeal RL (2002) Medication errors observed in 36 health care facilities. Arch Intern Med 162: 1897-1903. doi:10.1001/archinte.162.16.1897. PubMed: 12196090. 12196090 10.1001/archinte.162.16.1897

[28] Barker KN, Harris JA, Webster DB, Stringer JF, Pearson RE et al. (1984) Consultant evaluation of a hospital medication system: analysis of the existing system. Am J Hosp Pharm 41: 2009-2016. PubMed: 6496488. 6496488

[29] Barker KN, Mikeal RL, Pearson RE, Illig NA, Morse ML (1982) Medication errors in nursing homes and small hospitals. Am J Hosp Pharm 39: 987-991. PubMed: 7102695. 7102695

[30] Barker KN, Pearson RE, Hepler CD, Smith WE, Pappas CA (1984) Effect of an automated bedside dispensing machine on medication errors. Am J Hosp Pharm 41: 1352-1358. PubMed: 6465150. 6465150

[31] Borel JM, Rascati KL (1995) Effect of an automated, nursing unit-based drug-dispensing device on medication errors. Am J Health Syst Pharm 52: 1875-1879. PubMed: 8528848. 8528848 10.1093/ajhp/52.17.1875

[32] Bourlon S, Baronnet A, Provost S, Meunier P (2006) [Evaluation des erreurs médicamenteuses dans une unité de soins pédiatriques]. J Pharm Clin 25: 23-31.

[33] Bruce J, Wong I (2001) Parenteral drug administration errors by nursing staff on an acute medical admissions ward during day duty. Drug Saf 24: 855-862. doi:10.2165/00002018-200124110-00006. PubMed: 11665872. 11665872 10.2165/00002018-200124110-00006

[34] Chapuis C, Roustit M, Bal G, Schwebel C, Pansu P et al. (2010) Automated drug dispensing system reduces medication errors in an intensive care setting. Crit Care Med 38: 2275-2281. doi:10.1097/CCM.0b013e3181f8569b. PubMed: 20838333. 20838333 10.1097/CCM.0b013e3181f8569b

[35] Chua SS, Chua HM, Omar A (2010) Drug administration errors in paediatric wards: a direct observation approach. Eur J Pediatr 169: 603-611. doi:10.1007/s00431-009-1084-z. PubMed: 19823870. 19823870 10.1007/s00431-009-1084-z

[36] Conroy S, Appleby K, Bostock D, Unsworth V, Cousins D (2007) Medication errors in a children’s hospital. Paediatr Perinat Drug Ther 8: 18-25. doi:10.1185/146300907X167790.

[37] Dean B, Barber N (2000) The effects of a patients’ own drugs scheme on the incidence and severity of medication administration errors. Int J Pharm Practice 8: 209-216. doi:10.1111/j.2042-7174.2000.tb01007.x.

[38] DeYoung JL, Vanderkooi ME, Barletta JF (2009) Effect of bar-code-assisted medication administration on medication error rates in an adult medical intensive care unit. Am J Health Syst Pharm 66: 1110-1115. doi:10.2146/ajhp080355. PubMed: 19498127. 19498127 10.2146/ajhp080355

[39] Fahimi F, Ariapanah P, Faizi M, Shafaghi B, Namdar R et al. (2008) Errors in preparation and administration of intravenous medications in the intensive care unit of a teaching hospital: an observational study. Aust Crit Care 21: 110-116. doi:10.1016/j.aucc.2007.10.004. PubMed: 18387813. 18387813 10.1016/j.aucc.2007.10.004

[40] Font Noguera I, Climent C, Poveda Andrés JL (2008) [Quality of drug treatment process through medication errors in a tertiary hospital]. Farm Hosp 32: 274-279. doi:10.1016/S1130-6343(08)75946-4. PubMed: 19150042. 19150042

[41] Ford DG, Seybert AL, Smithburger PL, Kobulinsky LR, Samosky JT et al. (2010) Impact of simulation-based learning on medication error rates in critically ill patients. Intensive Care Med 36: 1526-1531. doi:10.1007/s00134-010-1860-2. PubMed: 20300731. 20300731 10.1007/s00134-010-1860-2

[42] Franklin BD, O’Grady K, Donyai P, Jacklin A, Barber N (2007) The impact of a closed-loop electronic prescribing and administration system on prescribing errors, administration errors and staff time: a before-and-after study. Qual Saf Health Care 16: 279-284. doi:10.1136/qshc.2006.019497. PubMed: 17693676. 17693676 10.1136/qshc.2006.019497 PMC2464943

[43] Franklin BD, O’Grady K, Parr J, Walton I (2006) Using the internet to deliver education on drug safety. Qual Saf Health Care 15: 329-333. doi:10.1136/qshc.2005.017608. PubMed: 17074868. 17074868 10.1136/qshc.2005.017608 PMC2565815

[44] Ghaleb MA, Barber N, Franklin BD, Wong IC (2010) The incidence and nature of prescribing and medication administration errors in paediatric inpatients. Arch Dis Child 95: 113-118. doi:10.1136/adc.2009.158485. PubMed: 20133327. 20133327 10.1136/adc.2009.158485

[45] Greengold NL, Shane R, Schneider P, Flynn E, Elashoff J et al. (2003) The impact of dedicated medication nurses on the medication administration error rate: a randomized controlled trial. Arch Intern Med 163: 2359-2367. doi:10.1001/archinte.163.19.2359. PubMed: 14581257. 14581257 10.1001/archinte.163.19.2359

[46] Han PY, Coombes ID, Green B (2005) Factors predictive of intravenous fluid administration errors in Australian surgical care wards. Qual Saf Health Care 14: 179-184. doi:10.1136/qshc.2004.010728. PubMed: 15933314. 15933314 10.1136/qshc.2004.010728 PMC1744016

[47] Hartley GM, Dhillon S (1998) An observational study of the prescribing and administration of intravenous drugs in a general hospital. Int J Pharm Pract 6: 38-45. doi:10.1111/j.2042-7174.1998.tb00914.x.

[48] Haw C, Stubbs J, Dickens G (2007) An observational study of medication administration errors in old-age psychiatric inpatients. Int J Qual Health Care 19: 210-216. doi:10.1093/intqhc/mzm019. PubMed: 17562662. 17562662 10.1093/intqhc/mzm019

[49] Helmons PJ, Wargel LN, Daniels CE (2009) Effect of bar-code-assisted medication administration on medication administration errors and accuracy in multiple patient care areas. Am J Health Syst Pharm 66: 1202-1210. doi:10.2146/ajhp080357. PubMed: 19535659. 19535659 10.2146/ajhp080357

[50] Ho CYW, Dean BS, Barber ND (1997) When do medication administration errors happen to hospital inpatients? Int J Pharm Practice 5: 91-96. doi:10.1111/j.2042-7174.1997.tb00891.x.

[51] Kelly J, Wright D, Wood J (2011) Medicine administration errors in patients with dysphagia in secondary care: a multi-centre observational study. J Adv Nurs 67: 2615-2627. doi:10.1111/j.1365-2648.2011.05700.x. PubMed: 21615463. 21615463 10.1111/j.1365-2648.2011.05700.x

[52] Lám J, Rózsa E, Kis Szölgyémi M, Belicza E (2011) [Survey of drug dispensing errors in hospital wards]. Orv Hetil 152: 1391-1398. doi:10.1556/OH.2011.29198. PubMed: 21846613. 21846613 10.1556/OH.2011.29198

[53] Le Grognec C, Lazzarotti A, Marie-Joseph DA, Lorcerie B (2005) [Medication errors resulting from drug preparation and administration]. Therapie 60: 391-399. doi:10.2515/therapie:2005057. PubMed: 16268439. 16268439 10.2515/therapie:2005057

[54] Means BJ, Derewicz HJ, Lamy PP (1975) Medication errors in a multidose and a computer-based unit dose drug distribution system. Am J Hosp Pharm 32: 186-191. PubMed: 1136964. 1136964

[55] O’Brodovich M, Rappaport P (1991) A study pre and post unit dose conversion in a pediatric hospital. Can J Hosp Pharm 44: 5-15, 50. PubMed: 10111736. 10111736

[56] Ozkan S, Kocaman G, Ozturk C, Seren S (2011) Frequency of pediatric medication administration errors and contributing factors. J Nurs Care Qual 26: 136-143. doi:10.1097/NCQ.0b013e3182031006. PubMed: 21135709. 21135709 10.1097/NCQ.0b013e3182031006

[57] Paoletti RD, Suess TM, Lesko MG, Feroli AA, Kennel JA et al. (2007) Using bar-code technology and medication observation methodology for safer medication administration. Am J Health Syst Pharm 64: 536-543. doi:10.2146/ajhp060140. PubMed: 17322168. 17322168 10.2146/ajhp060140

[58] Pastó-Cardona L, Masuet-Aumatell C, Bara-Oliván B, Castro-Cels I, Clopés-Estela A et al. (2009) [Incident study of medication errors in drug use processes: prescription, transcription, validation, preparation, dispensing and administering in the hospital environment]. Farm Hosp 33: 257-268. doi:10.1016/S1130-6343(09)72465-1. PubMed: 19775576. 19775576

[59] Poon EG, Keohane CA, Yoon CS, Ditmore M, Bane A et al. (2010) Effect of bar-code technology on the safety of medication administration. N Engl J Med 362: 1698-1707. doi:10.1056/NEJMsa0907115. PubMed: 20445181. 20445181 10.1056/NEJMsa0907115

[60] Pourrat X, Antier D, Doucet O, Duchalais A, Lemarié E et al. (2003) [Identification and analysis of errors in prescription, preparation and administration of drugs in intensive care, medicine and surgery at the University Hospital Center of Tours]. Presse Med 32: 876-882. PubMed: 12870395. 12870395

[61] Prot S, Fontan JE, Alberti C, Bourdon O, Farnoux C et al. (2005) Drug administration errors and their determinants in pediatric in-patients. Int J Qual Health Care 17: 381-389. doi:10.1093/intqhc/mzi066. PubMed: 16115809. 16115809 10.1093/intqhc/mzi066

[62] Raja Lope RJ, Boo NY, Rohana J, Cheah FC (2009) A quality assurance study on the administration of medication by nurses in a neonatal intensive care unit. Singapore Med J 50: 68-72. PubMed: 19224087. 19224087

[63] Reifsteck M, Swanson T, Dallas M (2006) Driving out errors through tight integration between software and automation. J Healthc Inf Manag 20: 35-39. PubMed: 17091788. 17091788

[64] Ridge KW, Jenkins DB, Noyce PR, Barber ND (1995) Medication errors during hospital drug rounds. Qual Health Care 4: 240-243. doi:10.1136/qshc.4.4.240. PubMed: 10156392. 10156392 10.1136/qshc.4.4.240 PMC1055333

[65] Rodriguez-Gonzalez CG, Herranz-Alonso A, Martin-Barbero ML, Duran-Garcia E, Mi Durango-Limarquez et al. (2012) Prevalence of medication administration errors in two medical units with automated prescription and dispensing. J Am Med Inform Assoc 19: 72-78. doi:10.1136/amiajnl-2011-000332. PubMed: 21890872. 21890872 10.1136/amiajnl-2011-000332 PMC3240760

[66] Ros H, de Vreeze-Wesselink E (2009) Reducing the number of dispensing errors by implementing a combination of a CPOE system and a bar-code-assisted dispensing system: the BAP concept. Eur J Hosp Pharm Science 15: 86-92.

[67] Rosati JR, Nahata MC (1983) Drug administration errors in pediatric patients. Qual Rev Bull 9: 34-35. PubMed: 6413923. 6413923

[68] Schneider MP, Cotting J, Pannatier A (1998) Evaluation of nurses’ errors associated in the preparation and administration of medication in a pediatric intensive care unit. Pharm World Sci 20: 178-182. doi:10.1023/A:1012087727393. PubMed: 9762730. 9762730 10.1023/a:1012087727393

[69] Schneider PJ, Pedersen CA, Montanya KR, Curran CR, Harpe SE et al. (2006) Improving the safety of medication administration using an interactive CD-ROM program. Am J Health Syst Pharm 63: 59-64. doi:10.2146/ajhp040609. PubMed: 16373466. 16373466 10.2146/ajhp040609

[70] Taxis K, Barber N (2004) Incidence and severity of intravenous drug errors in a German hospital. Eur J Clin Pharmacol 59: 815-817. doi:10.1007/s00228-003-0689-9. PubMed: 14586530. 14586530 10.1007/s00228-003-0689-9

[71] Taylor JA, Loan LA, Kamara J, Blackburn S, Whitney D (2008) Medication administration variances before and after implementation of computerized physician order entry in a neonatal intensive care unit. Pediatrics 121: 123-128. doi:10.1542/peds.2007-2022BBBB. PubMed: 18166565. 18166565 10.1542/peds.2007-0919

[72] Teixeira TC, de Cassiani SH (2010) [Root cause analysis: evaluation of medication errors at a university hospital]. Rev Esc Enferm U S P 44: 139-146. doi:10.1590/S0080-62342010000100020. 10.1590/s0080-62342010000100020 20394231

[73] Thur MP, Miller WA, Latiolais CJ (1972) Medication errors in a nurse-controlled parenteral admixture program. Am J Hosp Pharm 29: 298-304. PubMed: 5025259. 5025259

[74] Tisdale JE (1986) Justifying a pediatric critical-care satellite pharmacy by medication-error reporting. Am J Hosp Pharm 43: 368-371. PubMed: 3953597. 3953597

[75] Tissot E, Cornette C, Demoly P, Jacquet M, Barale F et al. (1999) Medication errors at the administration stage in an intensive care unit. Intensive Care Med 25: 353-359. doi:10.1007/s001340050857. PubMed: 10342507. 10342507 10.1007/s001340050857

[76] Tissot E, Cornette C, Limat S, Mourand JL, Becker M et al. (2003) Observational study of potential risk factors of medication administration errors. Pharm World Sci 25: 264-268. doi:10.1023/B:PHAR.0000006519.44483.a0. PubMed: 14689814. 14689814 10.1023/b:phar.0000006519.44483.a0

[77] van den Bemt PM, Fijn R, van der Voort PH, Gossen AA, Egberts TC et al. (2002) Frequency and determinants of drug administration errors in the intensive care unit. Crit Care Med 30: 846-850. doi:10.1097/00003246-200204000-00022. PubMed: 11940757. 11940757 10.1097/00003246-200204000-00022

[78] van Gijssel-Wiersma DG, van den Bemt PM, Walenbergh-van Veen MC (2005) Influence of computerised medication charts on medication errors in a hospital. Drug Saf 28: 1119-1129. doi:10.2165/00002018-200528120-00006. PubMed: 16329714. 16329714 10.2165/00002018-200528120-00006

[79] Westbrook JI, Rob MI, Woods A, Parry D (2011) Errors in the administration of intravenous medications in hospital and the role of correct procedures and nurse experience. Bmj Qual Saf 20: 1027–1034. doi:10.1136/bmjqs-2011-000089. PubMed: 21690248. 10.1136/bmjqs-2011-000089 PMC3228265 21690248

[80] Westbrook JI, Woods A, Rob MI, Dunsmuir WT, Day RO (2010) Association of interruptions with an increased risk and severity of medication administration errors. Arch Intern Med 170: 683-690. doi:10.1001/archinternmed.2010.65. PubMed: 20421552. 20421552 10.1001/archinternmed.2010.65

[81] Ghaleb MA, Barber N, Franklin BD, Yeung VW, Khaki ZF et al. (2006) Systematic review of medication errors in pediatric patients. Ann Pharmacother 40: 1766-1776. doi:10.1345/aph.1G717. PubMed: 16985096. 16985096 10.1345/aph.1G717

[82] Miller MR, Robinson KA, Lubomski LH, Rinke ML, Pronovost PJ (2007) Medication errors in paediatric care: a systematic review of epidemiology and an evaluation of evidence supporting reduction strategy recommendations. Qual Saf Health Care 16: 116-126. doi:10.1136/qshc.2006.019950. PubMed: 17403758. 17403758 10.1136/qshc.2006.019950 PMC2653149

[83] Fontan JE, Maneglier V, Nguyen VX, Loirat C, Brion F (2003) Medication errors in hospitals: computerized unit dose drug dispensing system versus ward stock distribution system. Pharm World Sci 25: 112-117. doi:10.1023/A:1024053514359. PubMed: 12840964. 12840964 10.1023/a:1024053514359

[84] The Cochrane Collaboration (2002) Publication bias Available: http://www.cochrane-net.org/openlearning/html/mod15.htm. Accessed 2012 Aug

[85] Perras C, Jacobs P, Boucher M, Murphy G, Hope J et al. (2009) Technologies to reduce errors in dispensing and administration of medication in hospitals: Clinical and economic analyses [Technology report number 121]. Ottawa: Canadian Agency for Drugs and Technologies in Health.

[86] McKibbon K, Lokker C, Handler S, Dolovich L, Holbrook A et al. (2011) Enabling medication management through health information technology. Evid Rep/Technol Assess No. 201. AHRQ Publication No 11-E008-EF. Rockville MD: Agency for Healthcare Research and Quality. PMC4781568 23126642

