# Supplementary material for: Drug Administration Errors in Hospital Inpatients: A Systematic Review
Source: PLoS One. 2013 Jun 20;8(6):e68856. doi: 10.1371/journal.pone.0068856 (PMC3688612; doi:10.1371/journal.pone.0068856)
Supplement: Table S2 — (DOC) [file pone.0068856.s004.doc]

**Table S2:** Characteristics of included studies

| Publication | Country | 1) Error rate 2) Error rate without WTE^a^ (if WTE studied or if WTE not included) | Number of observation^b^ | Structure | Observer | Period: day; hours | Number of error types | Intervention (for intervention studies) |
| --- | --- | --- | --- | --- | --- | --- | --- | --- |
| ***Barker*** *1962* | USA | 1) 16.3% (93/572)^c^ 2) 14.7%^c^ | 572 (DO) | 1 hospital, medical and surgical units, paediatric unit, obstetrical unit | 1 pharmacy resident | 2 consecutive 8h shifts, 136h observed; 3 shifts (day, evening, night) | 7 |  |
| ***Thur*** *1972* | USA | 1) 21.0% (21/100)^c^ 2) 21.0%^c^ | 100 (DO) | 1 hospital, 2 medical-surgical units | Pharmacists | 10-day period; 7 am - 11 pm | 4 |  |
| ***Means*** *1975* | USA | 1) 11.8%^c^ (245+70/2671) Multidose: 17.2%, unitdose: 9.7% 2) 4.7%^c^ (105+20/2671) | 2671 (DO) | 1 hospital, 3 adult medical units | NA | 60 days; 4h observation periods randomly scheduled | 5 |  |
| ***Rosati*** *1984* | USA | 1) 12.9% (28/217))^c^ 2) 5.5%^c^ | 217 (DO) | 1 hospital, various units | 2 pharmacists | NA | 5 |  |
| ***Wirtz*** *2003* | UK + Germany | 1) 29.4% (181/615)^c^ 2) 29.4%^c^ | 615 (DO) | 3 hospitals, surgical wards, intensive care units | 1 pharmacist | 6 consecutive days on each ward (36 days); peak times of drug administration (8 am, 12 am, 10 pm) | 11 |  |
| ***Taxis*** *2003* | UK | 1) 49.3% (212/430)^d^, 57.9% (249/430)^c^ 2) 49.3%^d^, 57.9%^c^ | 430 (DO) | 2 hospitals, 10 wards | 1 pharmacist | 6-10 consecutive days on each ward, including week-end (76 days); all times of drug rounds | 11 |  |
| ***Taxis*** *2004* | Germany | 1) 47.5% (58/122)^d^, 53.3% (65/122)^c^ 2) 47.5%^d^, 53.3%^c^ | 122 (DO) | 1 hospital, 1 surgical ward, 1surgical intensive care unit | 1 pharmacist | 6-7 consecutive days on each ward, including week-end, 2 to 3 drug rounds each day | 11 |  |
| ***Han*** *2005* | Australia | 1) 18.0% (124/687)^d^, 18.3% (126/687)^c^ 2) 18.0%^d^, 18.3%^c^ | 687 (DO) | 1 hospital, 3 surgical wards | 1 pharmacist | 4 week-period, except week-end; 9 am - 4 pm and 8 pm - 3 am | 10 |  |
| ***Teixeira*** *2010* | Brazil | 1) 8.6% (71/821)^c^ (WT delay unknown) 2) 6.6%^c^ | 821 (DO) | 1 hospital, 1 internal unit | NA | 30 days | 10 |  |
| ***Westbrook*** *2010* | Australia | 1) 25% (1067/4271)^d^, 28% (1196/4271)^c^ 2) 11.9%^c^ | 4271 (DO) | 2 hospitals, medical and surgical units | 3 researchers (registered nurses, physicians) | 340h, 164.75h; peak times: 7 am, 9.30 pm | 12 |  |
| ***Westbrook*** *2011* | Australia | 1) 63.9% (363/568)^d^, 72.4% (411/568)^c^ 2) 63.9%^d^, 72.4%^c^ | 568 (DO) | 2 hospitals, medical and surgical units | 3 researchers (registered nurses, physicians) | 340h, 164.75h; peak times: 7 am, 9.30 pm | 4 |  |
| ***Zribi Triki*** *2011* | Tunisia | 1) 100% (100/100)^d^, >100% (141/100)^c^ (WT delay unknown)  2) 100%^d^, >100%^c^ | 100 (DO) | 1 hospital, 1 orthopedic unit | 1 5-year pharmacy student | 1 month | 9 |  |
| ***Hynniman*** *1970* | USA | **Unit dose** 1) 3.5% (214/6061)^c^ (WT delay unknown) 2) 3.5%^c^. **Multidose** 1) 12.3% (664/5420)^c^ (WT delay unknown) 2) 11.5%^c^ | 6061 (TOE) 5420 (TOE) | Unit dose: 1 hospital, 4 medical units, 1 orthopedic unit. Multidose: 4 hospitals, 6 medical units, 1 surgical unit | pharmacy students | 2-week period (unit dose) | 6 |  |
| ***Barker*** *1982* | USA | 1) 17.4% (74/425)^c^ 2) 17.4%^c^ Results for hospitals only | 425 (TOE) | 10 hospitals, units | 12 nurses, 11 pharmacists | 1 day/site; 3h period (peak medication time) | 6 |  |
| ***Barker*** *1984-1* | USA | 1) 36.9% (745/2018)^c^ 2) 9.1%^c^ | 2018 (TOE) | 1 hospital, 1 medical unit, 2 surgical units, 1 continuing-care service nursing unit | 1 pharmacist | 17-day period, 34 five-hour periods on the day and evening shifts; 7.30 am - 12.30 am, 5.30 pm – 10 pm (surgical unit: 9.30 am - 2.30 pm) | 8 |  |
| ***Tisdale*** *1986* | USA | 1) 24.8% (147/592)^c^ 2) 8.8%^c^ | 592 (TOE) | 1 hospital, 1 intensive-care nursery and 1 paediatric intensive care unit | 1 pharmacist | 18 12h shifts; 5 day shifts/unit (7.30 pm - 7.30 pm); 4 night shifts/unit (7.30 pm - 7.30 am) | 8 |  |
| ***Dean*** *1995* | USA+UK | 1) 4.0% (147/3675)^c^ 2) 4.0%^c^ | 3675 (TOE) | 2 hospitals, 2 general medical-surgical units, 2 general medicine, 2 general surgery and 2 geriatric wards | 2 observers | UK: consecutive weekdays: 6 am, 8 am, 12 pm, 2 pm, 6 pm, 10 pm; USA: 8 am - 10.30 pm | 7 |  |
| ***Ridge*** *1995* | UK | 1) 3.5% (115/3312)^c^ 2) 3.5%^c^ | 3312 (TOE) | 1 hospital, 2 general surgery, 2 medicine for the elderly, 2 general medical wards | 2 pharmacists | at least 10 drug rounds over one week/ward; 4 or 5 scheduled rounds/day | 9 |  |
| ***Ho 1997*** | UK | 1) 5.5% (119/2170)^c^ 2) 5.5%^c^ | 2170 (TOE) | 1 hospital, 1 female care of elderly ward | 1 observer | 2 periods of 8 consecutive days (weekday, evening, weekend); 8 am, 12 pm, 5 pm/10 pm | 9 |  |
| ***Hartley*** *1998* | UK | 1) 78.6% (254/323)^c^ 2) 26.6%^c^ | 323 (TOE) | 1 hospital, 2 general surgical wards, 1 general medical ward | 1 pharmacist | 39 consecutive days; at varying times of the day: each administration round was observed until all administrations were complete | 10 |  |
| ***Schneider*** *1998* | Switzerland | 1) 26.9% (74/275)^c^ 2) 18.2%^c^ | 275 (TOE) | 1 hospital, 1 paediatric intensive care unit | 1 pharmacist | 10 week-period, twice a week; 8.30 am - 1.30 pm | 8 |  |
| ***Tissot*** *1999* | France | 1) 23.2% (132/568)^c^ 2) 21.7%^c^ | 568 (TOE) from methodology details article | 1 hospital, 1 intensive care unit | 2 pharmacy residents | 30 days during a 2-month period, except week-end/nights; 6 h per day (heaviest time periods) | 6 |  |
| ***Bruce*** *2001* | UK | 1) 25.2% (27/107)^c^ 2) 10.3%^c^ | 107 (TOE) | 1 hospital, 1 acute medical admissions ward | 1 pharmacist | 4 week-period, except week-end; 8 am - 4.30 pm | 12 |  |
| ***Calabrese*** *2001* | USA | 1) 3.3% (187/5744)^c^ 2) 2.8%^c^ | 5744 (TOE) | Hospitals, 5 surgical, medical and mixed intensive care units | Pharmacists | 3 months non-consecutively, except week-end; twice daily (morning, afternoon) | 10 |  |
| ***Barker*** *2002* | USA | 1) 16.4% (290/1765)^c^ 2) 9.9%^c^ Results for accredited and non accredited hospitals only | 1765 (TOE) | 24 hospitals: accredited hospitals, non accredited hospitals, units | 2 registered nurses, 2 licensed practical nurses, 2 pharmacy technicians per state | 81 observation days; 2h or until all doses due were administered | 8 |  |
| ***van den Bemt*** *2002* | the Netherlands | 1) 44.6% (104/233)^d^, 56.2% (131/233)^c^ 2) 33.0%^d^, 33.9%^c^ | 233 (TOE) | 2 hospitals, 2 intensive care units (mixed medical / surgical unit) | 1 pharmacist | 5 consecutive days, except week-end; 7 am - 10 pm | 8 |  |
| ***Pourrat*** *2003* | France | 1) 13.4% (219/1632)^d^, 13.4% (219/1632)^c^ 2) 8.3%^c^ | 1632 (TOE) | 3 hospitals, 1 surgery intensive care unit, 1 pneumology unit, 1 paediatric visceral surgery unit | 1 pharmacy resident | 79 days, 11 nights; 9 am - 6 pm, 8 pm - 2 am | 10 |  |
| ***Tissot*** *2003* | France | 1) 14.9% (78/523)^c^ 2) 11.1%^c^ | 523 (TOE) | 1 hospital, 1 geriatric unit, 1 cardiovascular-thoracic surgery unit | 1 pharmacist | 10 consecutive days/unit, except week-end (20 days); 9 am - 4 pm | 8 |  |
| ***Le Grognec*** *2005* | France | 1) 24% (52/217)^d^, 41.0% (89/217)^c^ 2) 34.6%^c^ | 217 (TOE) | 1 hospital, 1 internal medicine unit | Some 5-year pharmacy students | 32 days, except week-end; mornings | 5 |  |
| ***Lisby*** *2005* | Denmark | 1) 40.3% (166/412)^d^, 46.1% (190/412)^c^ 2) 41.7%^c^ | 412 (TOE) | 1 hospital, 1 medical ward, 1 surgical ward | 1 observer | 5 consecutive days/each ward (8h): 4 days in the daytime and one during evening shift; 2 nurses observed 4h/duty (8 am, 12 am, 5 pm, 10 pm) | 8 |  |
| ***Prot*** *2005* | France | 1) 27.2% (467/1719)^d^, 31.3% (538/1719)^c^ 2) 17.6%^d^, 20.1%^c^ | 1719 (TOE) | 1 hospital, 1 intensive care unit, 1 neonatal intensive care unit, 1 nephrology unit, 1 general paediatric unit | 12 5-year pharmacy students + 1 pharmacy resident | 271 days, except week-end; 2 consecutive hours in the mornings, all the nurses of the 4 units participated | 10 |  |
| ***Bourlon*** *2006* | France | 1) 51.8% (218/421)^d^, 76% (320/421)^c^ (WT delay unknown) 2) 72%^c^ | 421 (TOE) | 1 hospital, 1 paediatric-cardiological-endocrinology unit | 1 pharmacy resident | 20 days, except night; 7 am and 7 pm | 11 |  |
| ***Anselmi*** *2007* | Brasil | 1) 6.8% (184/2706)^c^ 2) 6.8%^c^ | 2706 (TOE) | 3 hospitals, 1 internal medicine, 1 surgery, 1 obstetrics, 1 paediatrics, 1 emergency | A research team for each hospital (including supervisor, observers: nurses, nursing students) | 35 days, except week-end; between 8 - 10 am, 11 - 12 am, 4 - 6 pm. The observer elaborated a list of the subjects that would be observed each day. | 8 |  |
| ***Conroy*** *2007* | UK | 1) 1.2% (9/752)^c^ (WT delay unknown) 2) 0.9%^c^ | 752 (TOE) | 1 hospital, 1 paediatric medical and 1 surgical wards, 1 paediatric intensive care unit, 1 neonatal intensive care unit, 1 emergency department | 1 pharmacist, 1 pharmacy technician | 6 week-period; 2 medicine rounds daily: 8 am and 12 am rounds > 6 pm and 10 pm | ? |  |
| ***Haw*** *2007* | UK | 1) 24.5% (349/1423)^d^, 25.9% (369/1423)^c^ (WT delay unknown) 2) 25.4%^c^ | 1423 (TOE) | 1 hospital, 2 long-stay wards for elderly mentally ill patients | 1 pharmacist | 2 week-period; each of the 4 routine daily drugs rounds: 8 am, 12 am, 6 pm, 10 pm | 11 |  |
| ***Fahimi*** *2008* | Iran | 1) 9.4% (380/4040)^c^ 2) 9.4%^c^ | 4040 (TOE) | 1 hospital, 1 intensive care unit | 1 pharmacist | 16 days; selection randomly from all shifts and during all hours (peak times of IV drug administration: 6 am, 9 am, noon, 2 pm, 6 pm, 10 pm, midnight) | ? |  |
| ***Font Noguera*** *2008* | Spain | 1) 2.1% (38/1789)^c^ (WT delay unknown) 2) 1.8%^c^ | 1789 (TOE) | 1 hospital, medical units | 6 fixed pairs of observers (trainee pharmacists and specialists in training in hospital pharmacy) | 8 am - 9 am | 17 |  |
| ***Chua*** *2009* | Malaysia | 1) 11.4% (127/1118)^d^, 12.1% (135/1118)^c^ 2) 8.7%^d^, 9%^c^ | 1118 (TOE) | 1 hospital, 1 haematology ward | 1 pharmacist | 8 consecutive weeks (15 working days), every Wednesday, Friday; 7.30 am - 9 pm | 11 |  |
| ***Pasto-Cardona*** *2009* | Spain | 1) 1.9% (171/8754)^c^ 2) 1.2%^c^ | 8784 (TOE) | 6 hospitals, medical and surgical units | 1 nurse experienced in charge/hospital | Daily; continually until the total observations has been reached | 13 |  |
| ***Chua*** *2010* | Malaysia | 1) 11.7% (100/857)^d^, 12.1% (104/857)^c^ 2) 7.8%^d^, 8.6%^c^ | 857 (TOE) | 1 hospital, 2 paediatric wards (general and oncology) | 1 pharmacist | 10 consecutive weeks, once a week; 7.30 am - 9 pm | 8 |  |
| ***Ghaleb*** *2010* | UK | 1) 19.1% (429/2249)^c^ (WT delay unknown) 2) 15.5%^c^ | 2249 (TOE) | 5 hospitals, 10 wards surgical, medical and intensive care paediatric | 1 pharmacist | 2 weeks/ward; WE, 8-12h/day | 12 |  |
| ***Gokhman*** *2011* | USA | 1) 98.4% (183/186)^d^, >100% (250/186)^c^ (WT delay unknown) 2) 98.4%^d^, >100%^c^ | 186 (TOE) | 1 hospital, 1 medical emergency unit | 1 pharmacist | NA | 13 |  |
| ***Lam*** *2011* | Hungary | 1) 14.2% (114/803)^d^, 16.7% (134/803)^c^ 2) 14.2%^d^, 16.7%^c^ | 803 (TOE) | 1 hospital, 1 neurology ward and 1 internal medicine ward | 1 nurse and 1 pharmacist (observation together) | 6 days/unit; 5 h/day | 8 |  |
| ***Kelly*** *2011* | UK | 1) 38.4% (817/2129)^c^ 2) 10.7%^c^ | 2129 (TOE) | 4 hospitals, care-of-the-elderly and stoke wards | 1 nurse | 4-month period, 8 shifts/ward; 2 shifts | 11 |  |
| ***Ozkan*** *2011* | Turkey | 1) 36.5% (855/2344)^c^ 2) 21.8%^c^ | 2344 (TOE) | 1 hospital, 1 paediatric unit | 2 nursing doctoral students | 50*12h observation periods; 1 day, 1 night shift/nurse | 9 |  |
| ***Rodriguez-Gonzalez*** *2011* | Spain | 1) 22% (509/2314)^c^ (WT delay unknown) 2) 20.7%^c^ | 2314 (TOE) | 1 hospital, 2 gastroenterology wards | 6 pharmacists and 5 nurses | 1 week | 16 |  |
| ***Schnell*** *1976* | Canada | **Before:** 1) 34.9% (4469/12803)^c^ (WT delay unknown) 2) 10.3%^c^ | 12803 (TOE) | 4 hospitals, 1 medical unit/ hospital | 1 observer/ hospital | 2 months/ hospital; 3 shifts | ? | Unit-dose dispensing system (before-after study) |
| ***Barker*** *1984-2* | USA | **Control group:** 1) 15.9% (139/873)^c^ 2) 6.8%^c^ | 873 (TOE) | 1 hospital, 1 general surgery unit | 1 pharmacist | 14 days; 5-hour peak medication periods: 7.30 am - 12.30 am, 5.30 pm - 10.30 pm (10h/ day) | 8 | Automated bedside dispensing machine (cross-over study) |
| ***O'Brodovich*** *1991* | Canada | **Before:** 1) 37.2% (105/282)^c^ 2) 10.3%^c^ | 282 (TOE) | 1 hospital, 2 paediatric medical wards | 4 pharmacists | 3 weeks, 100 hours, period of 2 hours; all nursing shifts | 9 | Unit-dose dispensing system (before-after study) |
| ***Borel*** *1995* | USA | **Before**: 1) 17.0% (148/873)^c^ (WT delay unknown) 2) 6.5%^c^ | 873 (TOE) | 1 hospital, 1 orthopaedic nursing unit, 1 general medical-surgical nursing unit, 1 general medical-surgical unit | 1 observer | 2 days per week; day and evening shifts | 10 | Automated bedside dispensing machine (before-after study) |
| ***Dean 2000*** | UK | **Before:** 1) 4.3% (119/3576)^c^ 2) 4.3% | 3576 (TOE) | 1 hospital, 1 vascular surgery ward, 1 renal medicine ward | 2 researchers | 17 days (weekday, weekend); 6 am, 12 pm, 6 pm, 10 pm | 8 | Introduction of patients’ own drugs |
| ***Greengold*** *2003* | USA | **General nurses:** 1) 6.9% (253/3661)^c^ 2) 6.9%^c^ | 3661 (TOE) | 2 hospitals, 8 units | 3 registered nurses, 2 pharmacy technicians | 2 contiguous 6-week blocks (3 weeks/unit), except week-end, 12 weeks; observation: 5 h/day | 8 | Dedicated medication nurses (randomized study) |
| ***van Gijssel-Wiersma*** *2005* | the Netherlands | **Before:** 1) 10.5% (118/1122)^c^ 2) 8.6%^c^ | 1122 (TOE) | 1 hospital, 1 internal medicine unit | 1 pharmacist | preintervention period: 02/2003, 3 weeks (introduction in 03/2003); observation: rounds (8 am + 3 pm) | 7 | Computerized medication charts (before-after study) |
| ***Franklin*** *2006* | UK | **Before:** 1) 6.9% (82/1188)^c^ 2) 6.9%^c^ | 1188 (TOE) | 1 hospital, 1 medical ward | 2 pharmacists | 4 weeks before intervention, 53 drug rounds | 16 | Education on drug safety (before-after study) |
| ***Reifsteck*** *2006* | USA | **Before:** 1) 23.5% (216/919)^c^ 2) 6.0% (55/919)^c^ | 919 (TOE) numerator extrapoled | Hospitals, Unit ? | Individuals trained | NA | 7 | Implementation of a medication system redesign |
| ***Schneider*** *2006* | USA | **Before (control + study groups):** 1) 9.5% (54/567)^c^ (WT delay unknown) 2) 8.3%^c^ | 567 (TOE) | 3 hospitals, medical units, surgical units | 2 observers | 2-week blocks/hospital, 6 weeks of observation; 4-h period during one shift/one week (baseline error) | 8 | Education on drug safety (randomized study) |
| ***Franklin*** *2007* | UK | **Before:** 1) 8.6% (141/1644)^c^ 2) 8.6%^c^ | 1644 (TOE) | 1 hospital, 1 general surgery ward | pharmacists | 2 week-period, including nights and week-end; 56 drug rounds (preintervention); drug rounds: 4 times/day | ? | Electronic prescribing and administration system  (before-after study) |
| ***Paoletti*** *2007* | USA | **Before (control + study groups):** 1) 20.1% (188/934)^c^ (WT delay unknown) 2) 4.8%^c^ | 934 (DO) | 1 hospital, 2 cardiac telemetry, 1 medical-surgical unit | 4 nurses | Peak workload periods | ? | Bar-code technology (before-after study) |
| ***Taylor*** *2008* | USA | **Before:** 1) 19.8% (50/253)^c^ 2) 9.9%^c^ | 253 (DO) | 1 hospital, 1 neonatal intensive care | research nurses | 53 days; 3h periods, during day, evening, night nursing shifts | 5 | Computerized physician order entry (before-after study) |
| ***Helmons*** *2009* | USA | **Before:** 1) 11.3% (142/1262)^c^ (WT delay unknown) 2) 8.9%^c^ | 1262 (TOE) | 1 hospital, 2 medical-surgical units, 2 intensive care units | 2 pharmacists, 6 pharmacy students | 1 month before implementation and 3 months after, including week-end; 9 am, 12 pm, 6 pm, 9 pm | 9 | Bar-code technology (before-after study) |
| ***DeYoung*** *2009* | USA | **Before:** 1) 19.7% (153/775)^d^, 22.5% (174/775)^c^ 2) 3.6%^c^ | 775 (TOE) | 1 hospital, 1 medical intensive care unit | pharmacy residents, pharmacists, 1 nurse | 4 consecutive days, 1 month before implementation; 7 am - 7 am, from Tuesday to Saturday | 7 | Bar-code technology (before-after study) |
| ***Raja*** ***Lope*** *2009* | Malaysia | **Before:** 1) 31.4% (59/188)^c^ 2) 0%^c^ | 188 (TOE) | 1 hospital, 1 neonatal intensive care unit | medical students | phase 1: 02/2005, phase 2: 02/2006 one week; 24-h period | 9 | Education on drug safety (randomized study) |
| ***Ros*** *2009* | The Netherlands | **Before:** 1) 7.2% (319/4457)^c^ 2) 3.1%^c^ | 4457 (TOE) | 1 hospital, 1 neurology unit | NA | 21 days, week-end included; 8 am, 12 pm, 5 pm | 8 | Implementation of a CPOE system and of bar-code-assisted dispensing of drugs |
| ***Chapuis*** *2010* | France | **Before (control + study groups):** 1) 3.9% (133/3391)^c^ 2) 3.2%^c^ | 3391 (TOE) (678+898+695+1120) | 1 hospital, 2 medical intensive care units | 1 pharmacist | 2 months; 3-4h, 4 days/week including week-end, night | 11 | Implementation of an automated drug dispensing system |
| ***Ford*** *2010* | USA | **Before:** 1) 23.8% (75/315)^c^ 2) 23.8%^c^ exclusion of prescribing errors | 315 (TOE) | 1 hospital, 1 coronary critical care unit and 1 medical intensive care unit | 2 pharmacists | several 4h sessions; 8 am-12 pm or 21 pm-4 pm | 13 exclusion of prescribing errors | Educational sessions on medication administration errors |
| ***Poon*** *2010* | USA | **Before:** 1) 28.3% (1905/6723)^c^ 2) 11.6%^c^ (779 errors when summing errors types, it differs from 776 presented in table 2) | 6723 (TOE) | 1 hospital, 35 medical, surgical, intensive care units | Research nurses | 4h, 7 am-3 pm | 7 | Bar-code technology |

^a^ WTE: wrong time errors

^b^ DO: doses observed, TOE: Total Opportunity for Errors

^c^ Error rate calculated as the number of errors/denominator

^d^ Error rate calculated as the number of administrations with at least one error/denominator

NA: Data Not Available
