# Supplementary material for: Drug Administration Errors in Hospital Inpatients: A Systematic Review
Source: PLoS One. 2013 Jun 20;8(6):e68856. doi: 10.1371/journal.pone.0068856 (PMC3688612; doi:10.1371/journal.pone.0068856)
Supplement: Table S3 — (DOC) [file pone.0068856.s005.doc]

**Table S3:** **Clinical impact of administration errors**

| Publication | Method for clinical impact evaluation | Clinical impact determined by | Fatal, n (%) | Life-threating, n (%) | Significant, n (%) | Minor or no impact, n (%) | Comments^a^ |
| --- | --- | --- | --- | --- | --- | --- | --- |
| Calabrese 2001 | Derived from NCCMERP | NA | 0/187 errors (0) | NA | 2/187 errors (1) | 185/187 errors (99) | Categories B- D: no harm, reclassified as “minor or no impact”, Categories E-H: harm, reclassified as “significant”, Category I: death, reclassified as “fatal” |
| van den Bemt 2002 | Derived from NCCMERP | Two pharmacists | 0/131 errors (0) | 0/131 errors (0) | 0/131 errors (0) | 131/131 errors (100) | Categories A- C: no harm, reclassified as “minor or no impact”, Categories D1-D3: harm, reclassified as “significant”, Category D4: harm, reclassified as “life-threatening”, Category E: death, reclassified as “fatal” |
| van Gijssel-Wiersma 2005 | Derived from NCCMERP | Two pharmacists (consensus) | 0/ 118 errors (0) | NA | 41/118 errors (35) | 77/118 errors (65) | Categories A- B: no harm, reclassified as “minor or no impact”, Categories C-D: harm, reclassified as “significant”, Category E: death, reclassified as “fatal” |
| Pasto-Cardona 2009 | Derived from NCCMERP | NA | 0/171 errors (0) | 0/171 errors (0) | 3/171 errors (2) | 168/171 errors (98) | Categories A- D: no harm, reclassified as “minor or no impact”, Categories E-G: harm, reclassified as “significant”, Category H: affected the life of the patient, reclassified as “life-threatening”, Category I: death, reclassified as “fatal” |
| Tissot 1999 | Categories (Folli 1987) | One physician | 0/132 errors (0) | 26/132 errors (20) | 55/132 errors (42) | 51/132 errors (39) | “four categories of clinical significance: fatal (resulting in death), life-threatening (resulting in serious side-effects requiring corrective treatment and increasing the length of hospitalization), significant (resulting in an increase in patient monitoring but without corrective treatment) and minor (no clinical relevance).” |
| Tissot 2003 | Categories (Folli 1987) | Multidisciplinary team (physicians, pharmacists) | 0/78 errors (0) | 8/78 errors (10) | 20/78 errors (26) | 50/78 errors (64) | “four categories: fatal (resulting in death), life-threatening (resulting in serious side effects requiring corrective treatment and/or increasing the length of hospitalization), significant (resulting in an increase in patient monitoring but without corrective treatment) or minor (no clinical relevance).” |
| Pourrat 2003 | Categories (Bayliff 1990) | Pair of pharmacist and physician | NA | 0/219 errors (0) | 42/219 errors (19) | 177/219 errors (81) | 4 categories: Impact 0: no impact (11%), Impact 1: minor impact (70%), reclassified as “minor or no impact”, Impact 2: significant impact (19%), reclassified as “significant”, Impact 3: potentially fatal (0%), reclassified as “life-threatening” |
| Le Grognec 2005 | Categories | One physician | 0/89 errors (0) | 3/89 errors (3) | 3/89 errors (3) | 83/89 errors (93) | Classification in 4 categories: fatal, serious, significant and minor |
| Chua 2009 | Categories (Stubbs 2004) | A clinician and a pharmacist (Kappa statistics, consensus) | NA | 14/135 errors (10) | 44/135 errors (33) | 77/135 errors (57) | « four categories based on that used by Stubbs et all, for prescribing errors: Grade (1) probably clinically insignificant, (2) minimal clinical significance, (3) definitely clinically significant and could cause patient harm and (4) potentially life-threatening.”  Grades 1 et 2 were reclassified as “minor or no impact” |
| Chua 2010 | Categories (Stubbs 2004) | A clinician and two pharmacists (consensus) | NA | 0/104 errors (0) | 42/104 errors (40) | 62/104 errors (60) | cf Chua 2009 J Clin Pharm Ther |
| Poon 2010 | Categories (Folli) | NA | NA | 2/779 errors (0.3) | 211/779 errors (27) | 566/779 errors (73) | 213 errors with severity/779 for non-timing errors |
| Rodriguez-Gonzalez 2012 | Categories (Ruiz Jarabo taxonomy) | Observer and 2 senior pharmacists | NA | NA | 2/479 errors (0.4) | 469/479 errors (98) | 1.6% of cases (n=8), potential clinical severity could not be assessed |
| Taxis 2003 | Scale (Dean 1999) | One physician, one nurse and two pharmacists | NA | 3/249 errors (1) | 144/249 errors (58) | 102/249 errors (41) | “Minor scores below to 3 suggested a minor outcome, scores of 3-7 a moderate outcome and scores above 7 a severe outcome.” Potentially minor errors: 41%, potentially moderate errors: 58%, potentially severe errors: 1% |
| Taxis 2004 | Scale (Dean 1999) | One physician, one nurse and one pharmacist | NA | 4/65 errors (6) | 39/65 errors (60) | 22/65 errors (34) | “Minor scores below to two suggested a minor outcome, scores of two to six a moderate outcome and scores above six a severe outcome.” Potentially minor errors: 34%, potentially moderate errors: 60%, potentially severe errors: 6% |
| Lisby 2005 | Scale | Two doctors in each ward and three pharmacists | 2/166 errors (1) | 33/166 errors (20) | 53/166 errors (32) | 77/166 errors (46) | Potential clinical consequences |
| Haw 2007 | Scale (Stubbs 2006) | Three researchers (consensus) | 0/369 errors (0) | NA | 1/369 errors (0.3) | 282/369 errors (76) | “Error severity was rated on the following five-point scale.” “Grade 1-errors or omissions of doubtful or negligible importance, Grade 2-errors or omissions likely to result in minor adverse effects or worsening condition, Grade 3-errors or omissions likely to result in serious effects or relapse, Grade 4-errors or omissions likely to result in fatality, Grade X-unrateable (due to lack of clinical and other information.” Grades 1 and 2 reclassified as “minor or no impact” Grade X unrateable: 86/369 errors (23%) |
| Westbrook 2010 | Scale (5-point scale severity Assessment code scale) | Two researchers | 0/1196 errors (0) | 9/1196 errors (1) | 106/1196 errors (9) | 1081/1196 errors (90) | Most errors (79.3%) were rated as insignificant (severity level 1). Only 115 (2.7%) were rated as major (106 at level 3 and 9 at level 4). None were rated at level 5. |
| Lam 2011 | Worst case scenario | Two pharmacists, Kappa statistic | NA | NA | (13) | (87) |  |

^a^Derived from publication results or direct citations from the text

NA: Data Not Available
